# Novel rare variant associations with late‐life cognitive performance

**DOI:** 10.1002/alz.095364

**Published:** 2025-01-09

**Authors:** Alexandra N. Regelson, Derek B. Archer, Alaina Durant, Shubhabrata Mukherjee, Michael L. Lee, Seo‐Eun Choi, Phoebe Scollard, Emily H. Trittschuh, Jesse Mez, William S. Bush, Amanda B Kuzma, Michael L. Cuccaro, Carlos Cruchaga, Lindsay A. Farrer, Li‐San Wang, Gerard D. Schellenberg, Richard Mayeux, Walter A. Kukull, C. Dirk Keene, Andrew J. Saykin, Sterling C. Johnson, Corinne D. Engelman, David A. Bennett, Lisa L. Barnes, Eric B Larson, Kwangsik Nho, Alison M. Goate, Alan E. Renton, Edoardo Marcora, Brian Fulton‐Howard, Tulsi Patel, Shannon L. Risacher, Anita L. DeStefano, Julie A. Schneider, Mohamad Habes, Sudha Seshadri, Claudia L Satizabal, Pauline Maillard, Arthur W. Toga, Karen Crawford, Duygu Tosun, Jeffery M. Vance, Elizabeth Mormino, Charles S. DeCarli, Thomas J. Montine, Gary Beecham, Sarah A Biber, Philip L. De Jager, Badri N. Vardarajan, Annie J. Lee, Adam M. Brickman, Christiane Reitz, Jennifer J. Manly, Qiongshi Lu, Miguel Arce Rentería, Yuetiva Deming, Margaret A Pericak‐Vance, Jonathan L. Haines, Paul K. Crane, Timothy J. Hohman, Logan C. Dumitrescu

**Affiliations:** ^1^ Vanderbilt Memory & Alzheimer’s Center, Vanderbilt University Medical Center, Nashville, TN USA; ^2^ Vanderbilt Genetics Institute, Vanderbilt University Medical Center, Nashville, TN USA; ^3^ Department of Medicine, University of Washington, Seattle, WA USA; ^4^ Department of Psychiatry and Behavioral Sciences, University of Washington School of Medicine, Seattle, WA USA; ^5^ VA Puget Sound Health Care System, Seattle, WA USA; ^6^ Department of Neurology, Boston University Chobanian & Avedisian School of Medicine, Boston, MA USA; ^7^ Cleveland Institute for Computational Biology, Department of Population and Quantitative Health Sciences, Case Western Reserve University, Cleveland, OH USA; ^8^ Penn Neurodegeneration Genomics Center, Department of Pathology and Laboratory Medicine, Perelman School of Medicine, University of Pennsylvania, Philadelphia, PA USA; ^9^ John P. Hussman Institute for Human Genomics, University of Miami Miller School of Medicine, Miami, FL USA; ^10^ Department of Psychiatry, Washington University School of Medicine, St. Louis, MO USA; ^11^ NeuroGenomics and Informatics Center, Washington University School of Medicine, St Louis, MO USA; ^12^ Department of Medicine (Biomedical Genetics), Boston University Chobanian & Avedisian School of Medicine, Boston, MA USA; ^13^ Department of Biostatistics, Boston University School of Public Health, Boston, MA USA; ^14^ University of Pennsylvania, Philadelphia, PA USA; ^15^ Columbia University, New York, NY USA; ^16^ Taub Institute for Research on Alzheimer’s Disease and the Aging Brain, Columbia University, New York, NY USA; ^17^ The Institute for Genomic Medicine, Columbia University Medical Center, New York, NY USA; ^18^ Department of Epidemiology, School of Public Health, University of Washington, Seattle, WA USA; ^19^ Department of Laboratory Medicine and Pathology, University of Washington, Seattle, WA USA; ^20^ Department of Medical and Molecular Genetics, Indiana University School of Medicine, Indianapolis, IN USA; ^21^ Indiana Alzheimer’s Disease Research Center, Indiana University School of Medicine, Indianapolis, IN USA; ^22^ Department of Radiology and Imaging Sciences, Indiana University School of Medicine, Indianapolis, IN USA; ^23^ Alzheimer’s Disease Research Center, University of Wisconsin School of Medicine and Public Health, Madison, WI USA; ^24^ Rush Alzheimer’s Disease Center, Rush University Medical Center, Chicago, IL USA; ^25^ Ronald M. Loeb Center for Alzheimer’s Disease, Friedman Brain Institute, Icahn School of Medicine at Mount Sinai, New York, NY USA; ^26^ Glenn Biggs Institute for Alzheimer’s & Neurodegenerative Diseases, University of Texas Health Sciences Center at San Antonio, San Antonio, TX USA; ^27^ Department of Neurology and Center for Neuroscience, University of California, Davis, CA USA; ^28^ Laboratory of Neuro Imaging, USC Stevens Neuroimaging and Informatics Institute, Keck School of Medicine, University of Southern California, Los Angeles, CA USA; ^29^ Department of Radiology and Biomedical Imaging, University of California, San Francisco, San Francisco, CA USA; ^30^ Department of Neurology and Neurological Sciences, Stanford University School of Medicine, Stanford, CA USA; ^31^ Department of Neurology & Imaging of Dementia and Aging Laboratory, University of California, Davis, Davis, CA USA; ^32^ Department of Pathology, Stanford University School of Medicine, Stanford, CA USA; ^33^ Dr. John T. Macdonald Foundation Department of Human Genetics, University of Miami Miller School of Medicine, Miami, FL USA; ^34^ National Alzheimer’s Coordinating Center, University of Washington, Seattle, WA USA; ^35^ Center for Translational & Computational Neuroimmunology, Columbia University Irving Medical Center, New York, NY USA; ^36^ Department of Neurology, Columbia University Medical Center, New York, NY USA; ^37^ The Gertrude H. Sergievsky Center, College of Physicians and Surgeons, Columbia University, New York, NY USA; ^38^ Department of Neurology, Vagelos College of Physicians and Surgeons, Columbia University, and the New York Presbyterian Hospital, New York, NY USA; ^39^ G. H. Sergievsky Center, Vagelos College of Physicians and Surgeons, Columbia University, New York, NY USA; ^40^ Department of Statistics, University of Wisconsin‐Madison, Madison, WI USA; ^41^ Taub Institute for Research on Alzheimer’s Disease and the Aging Brain, Columbia University Medical Center, New York, NY USA; ^42^ Department of Neurology, Vagelos College of Physicians and Surgeons, Columbia University, New York, NY USA; ^43^ Alzheimer’s Disease Research Center, University of Wisconsin‐Madison School of Medicine and Public Health, Madison, WI USA

## Abstract

**Background:**

Despite evidence that Alzheimer’s disease (AD) is highly heritable, there remains substantial “missing” heritability, likely due in part to the effect of rare variants and to the past reliance on case‐control analysis. Here, we leverage powerful endophenotypes of AD (cognitive performance across multiple cognitive domains) in a rare variant analysis to identify novel genetic drivers of cognition in aging and disease.

**Method:**

We leveraged 8 cohorts of cognitive aging with whole genome sequencing data from the AD Sequencing Project to conduct rare variant analyses of multiple domains of cognition (N = 9,317; mean age = 73; 56% female; 52% cognitively unimpaired). Harmonized scores for memory, executive function, and language were derived using confirmatory factor analysis models. Participants genetically similar to the 1000Genomes EUR reference panel were included in analysis. Variants included in the analysis had a minor allele frequency < 0.01, a minor allele count of ≥ 10, and were annotated as a high or moderate impact SNP using VEP. Associations of baseline scores in each cognitive domain were performed using SKAT‐O, including 92,905 rare variants among 16,243 genes. All tests were adjusted for sex, baseline age, sequencing center and platform, and genetic principal components. Correction for multiple comparisons was completed using the Benjamini‐Hochberg false discovery rate (FDR) procedure.

**Result:**

*APOE* was associated with baseline memory, language, and executive function, though only memory survived multiple‐test correction (p.FDR = 0.001). Outside of *APOE*, *ITPKB* was associated with baseline executive function (p.FDR = 0.048). *AKTIP*, *SHCBP1L*, and *CCNF* showed nominal associations with multiple domains of cognition that did not survive correction for multiple comparisons (p.FDRs<0.07).

**Conclusion:**

These results highlight novel rare variants associated with cognition. *IPTKB* is an AGORA nominated gene target for potential AD treatment. It is important in the regulation of immune cells and displays higher expression in the cortex of AD patients compared to controls. *CCNF* and *AKTIP* are brain eQTLs and have differential RNA expression in AD brains. Previously, variants in *AKTIP* have been associated with educational attainment, intelligence, and memory, while variants in *CCNF* have been associated with neuritic plaques and neurofibrillary tangles. Future analyses will incorporate longitudinal cognition and expand into additional populations.

